# Modeling the Impact of an Indoor Air Filter on Air Pollution Exposure Reduction and Associated Mortality in Urban Delhi Household

**DOI:** 10.3390/ijerph16081391

**Published:** 2019-04-17

**Authors:** Jiawen Liao, Wenlu Ye, Ajay Pillarisetti, Thomas F. Clasen

**Affiliations:** 1 Department of Environmental Health, Rollins School of Public Health, Emory University, Atlanta, GA 30322, USA; wenlu.ye@emory.edu (W.Y.); thomas.f.clasen@emory.edu (T.F.C.); 2 Environmental Health Sciences, School of Public Health, University of California, Berkeley, CA 94720, USA; ajaypillarisetti@berkeley.edu

**Keywords:** fine particulate matters (PM_2.5_), air filter, indoor air quality, CONTAM program, air exchange rate, health impact

## Abstract

Indoor exposure to fine particulate matter (PM_2.5_) is a prominent health concern. However, few studies have examined the effectiveness of long-term use of indoor air filters for reduction of PM_2.5_ exposure and associated decrease in adverse health impacts in urban India. We conducted 20 simulations of yearlong personal exposure to PM_2.5_ in urban Delhi using the National Institute of Standards and Technology’s CONTAM program (NIST, Gaithersburg, MD, USA). Simulation scenarios were developed to examine different air filter efficiencies, use schedules, and the influence of a smoker at home. We quantified associated mortality reductions with Household Air Pollution Intervention Tool (HAPIT, University of California, Berkeley, CA, USA). Without an air filter, we estimated an annual mean PM_2.5_ personal exposure of 103 µg/m^3^ (95% Confidence Interval (CI): 93, 112) and 137 µg/m^3^ (95% CI: 125, 149) for households without and with a smoker, respectively. All day use of a high-efficiency particle air (HEPA) filter would reduce personal PM_2.5_ exposure to 29 µg/m^3^ and 30 µg/m^3^, respectively. The reduced personal PM_2.5_ exposure from air filter use is associated with 8–37% reduction in mortality attributable to PM_2.5_ pollution in Delhi. The findings of this study indicate that air filter may provide significant improvements in indoor air quality and result in health benefits.

## 1. Introduction

Air pollution has been linked to increased risk of numerous diseases, including respiratory tract infections [1], exacerbations of inflammatory lung conditions [2,3], cardiac events [3], cancer [4], and low birth weight [5], and is regarded as one of the largest global health risk factors [6,7]. In India, 1.24 million (95% CI: 1.09–1.39) deaths in 2017 were attributable to air pollution, which was 12.5% of the total deaths in the country. Among these, 0.67 million (95% CI: 0.55–0.79) were attributed to ambient air pollution (AAP) and 0.48 million (95% CI: 0.39–0.58) were attributed to household air pollution (HAP) [8]. Studies of fine particulate matter (PM_2.5_) predominate air pollution research, mainly due to the detrimental health effects and high concentrations of PM_2.5_ in both indoor and outdoor environments [9,10,11]. 

While solid fuel combustion emits high levels of HAP in rural households [12,13], the combination of HAP generated from both local and regional sources plus the AAP generated from industrial activities, the power sector, and transportation elevates the risk in urban settings [14,15]. This is especially evident in India where four of the five cities with the highest ambient PM_2.5_ levels worldwide are located [16]. The annual population-weighted mean exposure to ambient PM_2.5_ in India was 89.9 µg/m^3^ (95% uncertainty interval (UI) 67.0–112.0) in 2017, which was one of the highest in the world. Among all Indian states, Delhi had the highest annual population-weighted mean ambient PM_2.5_ level in 2017 (209.0 µg/m^3^ (95% UI 120.9–339.5)), far beyond the limit recommended by the National Ambient Air Quality Standards in India [8].

In many parts of the world, both ambient and indoor PM_2.5_ contributed to personal exposure to PM_2.5_ [14]. Personal exposure to PM_2.5_ is determined by the PM_2.5_ concentrations in indoor and outdoor environments, as well as the time-activity patterns of the exposed individual [9]. Many national surveys and studies have shown that Indians spend most of their time in the indoor environment [9,17,18], arguably making indoor spaces the most important environment in which to mitigate PM_2.5_ exposures. PM_2.5_ concentrations in the indoor environment are influenced by various factors, including indoor emission sources, outdoor PM_2.5_ levels, airflows into the home environment, and removal of the PM_2.5_ inside the home [19]. Many of these factors are dynamic and vary by time; thus, indoor PM_2.5_ levels and exposure to PM_2.5_ levels change over time with distinct diurnal and seasonal patterns that are modulated by individual and household level behaviors.

Previous evidence in both developed [20,21] and developing countries [22,23] show that indoor PM_2.5_ concentrations can be reduced effectively and substantially by using air filters. Such air filtration has also been shown to have cardiovascular and pulmonary health benefits [22,24], including reduced asthma symptoms and inflammation and improved airway mechanics [25]. Several studies in India have investigated the effectiveness of HAP interventions on personal exposure to PM_2.5_ and health benefits [26,27,28], and studies in urban Indian cities suggest that an air filter intervention alone cannot reduce personal exposure to PM_2.5_ to the interim target guideline recommended by the World Health Organization (WHO) of 35 μg/m^3^ [7,23]. This is mainly due to the dynamics of airborne contaminants and continued infiltration of ambient air pollution [29]. In addition, the existing air filter intervention studies only provided data on short-term health responses due to reduced exposures, such as changes in cardiovascular biomarker level [22,30] or pulmonary functions of children with asthma [21,24,31]. The profile of long-term air pollution reduction and health benefits associated with air filter use have not been assessed or evaluated.

Models that simulate indoor concentrations and personal exposure to PM_2.5_ levels can be used to examine the changes in personal exposure to PM_2.5_ and estimate the effectiveness of potential pollution reduction strategies [29,32,33,34]. One of the validated simulation tools that has been widely applied is the CONTAM program (National Institute of Standards and Technology NIST, Gaithersburg, MD, USA, https://www.nist.gov/services-resources/software/contam), a multi-zone computer program that simulates airflow between each zone and estimates contaminant concentrations or personal exposures [35]. CONTAM has been applied extensively to assess indoor air quality in existing residential buildings, and to evaluate the effectiveness of indoor air quality control interventions in residential homes in United States cities [32,34]. The advantage of using the CONTAM simulation program is that it is a publicly available program and can simulate time-resolved ventilation rates, pollutant concentrations, and personal exposure levels based on air pollutants emission, decay rates, as well as climate and ambient air pollution levels [36]. To our knowledge, this is the first study to utilize CONTAM to assess the effectiveness of an indoor air quality intervention in residential household in developing countries.

In this study, we used CONTAM to simulate PM_2.5_ exposure over a one-year period for an occupant living in a typical residential apartment in urban Delhi, the dense urban area with the highest population-weight ambient PM_2.5_ concentration in 2017 [8]. We assessed the effectiveness of air filters at different efficiency levels and under different user scenarios. We further estimated the effects of reduced PM_2.5_ exposure on mortality using a customized version of the Household Air Pollution Intervention Tool (HAPIT v.3.1, University of California, Berkeley, CA, USA https://hapit.org) [37].

## 2. Materials and Methods 

### 2.1. Study Overview

**Figure 1:**
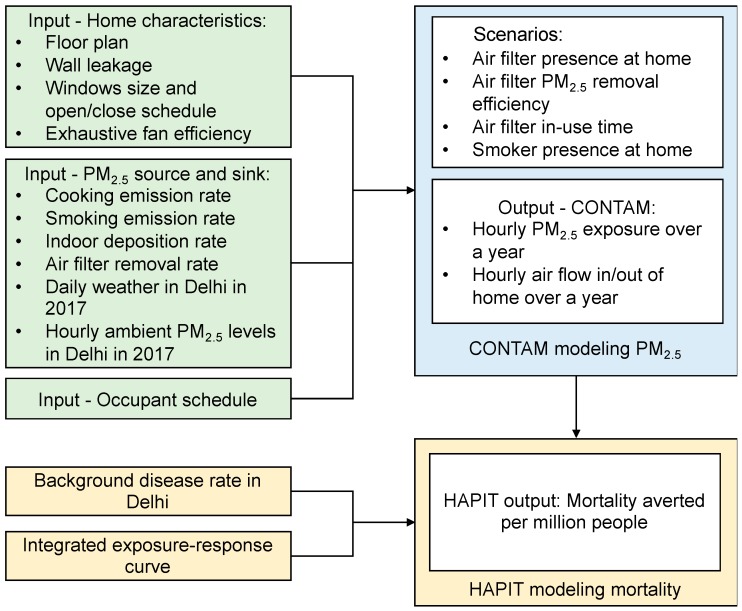
Flowchart of the study procedure and model input/output.

We used CONTAM (version 3.2) to estimate indoor air pollution concentrations and concentration reductions from an air filter running under a range of scenarios. A flowchart summarizing the study procedures is illustrated in Figure 1. We identified and defined the characteristics of a typical home in Delhi, including major PM_2.5_ sources and sinks and occupant schedules, and incorporated daily weather data and hourly ambient air pollution levels in 2017 as inputs for CONTAM simulations (Table 1). Then, we estimated annual indoor PM_2.5_ levels and indoor PM_2.5_ exposure for an occupant under 20 constructed scenarios based on the factorial design of the following factors: Presence/absence of an air filter at home, the efficiency of the air filter, air filter use duration, and the presence/absence of an active smoker at home. Finally, we quantified the potential health benefit associated with exposure reduction resulted from air filter use based on the indoor PM_2.5_ exposure differences.

### 2.2. Simulated Indoor Environment

**Figure 2:**
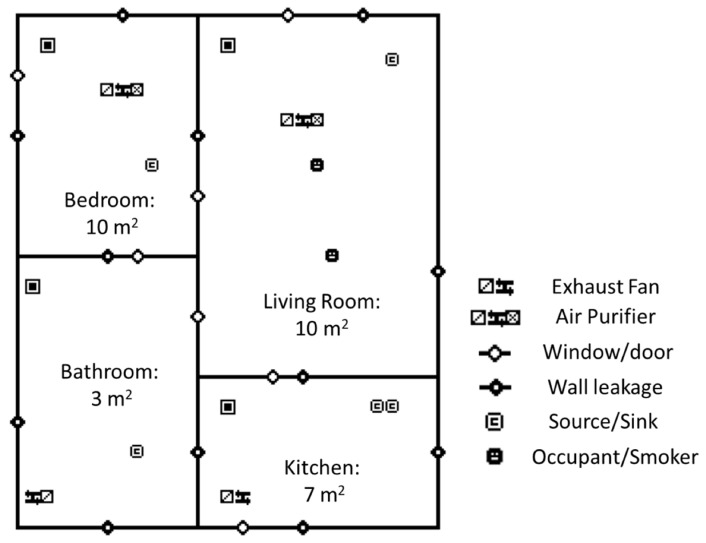
Simulated floor plan and corresponding CONTAM schematic.

We used CONTAM version 3.2 to estimate the reduction of exposure to indoor PM_2.5_ concentrations from an air filter running under a range of scenarios. We defined a house template in CONTAM to simulate an apartment typical of urban Delhi—one that is located on the 1st floor of a building, naturally ventilated, and contains a bedroom, living room, kitchen and bathroom, with a total area of 30 m^2^ (Figure 2). This is one of the most common apartment floor plans in the four main cities of India [38]. The apartment has a daily air exchange rate (AER) ranging from 0.3 to 4.5/h, with annual mean of 1.5/h. Detailed house characteristics, air exchange schedules, and the references for the assumptions are presented in Table 1.

### 2.3. Contaminant Sources and Sinks

Major sources of indoor PM_2.5_ include cooking, smoking, and outdoor infiltration through windows and wall leakages. Indoor PM_2.5_ removal mechanisms include deposition, exfiltration to outdoor air through exhaust fan(s), windows and wall leakage, and removal of PM_2.5_ by a portable air filter device, which is commonly seen on the Indian market [23]. For cooking emissions, we used the PM_2.5_ emission rate from the liquefied petroleum gas (LPG) stove use instead of biomass use [39], since Delhi has very high LPG coverage and traditional biomass only accounts for a small proportion of cooking energy in urban India [40,41]. We estimated the PM_2.5_ emission rate for smoking cigarettes at 0.33 mg/min [31] and with an average frequency of 8 cigarettes per day [42]. In addition, we assumed a PM_2.5_ deposition rate of 0.19/h [31] and simulated PM_2.5_ removal by the portable air filter devices with different minimal efficiency removal values (MERV), corresponding to different PM_2.5_ removal rates [43]. Table 2 summarizes the emission and removal rates used for each PM_2.5_ source and sink.

The hourly ambient PM_2.5_ data in urban Delhi were obtained from all available air pollution monitoring stations of the Central Pollution Control Board between 01/01/2017 and 12/31/2017 [46]. While India has expanded ground air pollution monitoring in recent years [47], only 9 air pollution monitoring stations are available in Delhi over the whole year of 2017 [46]. Table S1 lists the name, latitude, and longitude of all air pollution monitoring stations used in this study. The maximum distance between the monitoring stations is less than 10 km. We constructed hourly PM_2.5_ concentrations in 2017 over 365 days from all ambient air pollution monitoring stations (N = 9) to represent hourly PM_2.5_ concentrations in the urban Delhi region. Figure S1 shows the map of the Delhi region and the location of ambient air monitoring stations used in this study.

Hourly weather parameters (including temperature, wind speed, wind direction, relative humidity, and pressure) for a typical meteorological year were obtained from Energy Plus (United States Department of Energy) [45] to represent normal weather conditions in Delhi. All of these parameters are inputs to model the hourly transient airflow and PM_2.5_ concentrations in indoor settings.

### 2.4. Simulation Scenarios

We conducted a factorial design to allow inclusion of scenarios that might be observed in a typical apartment in urban Delhi. The key dimensions in our CONTAM factorial design were (i) air filter types (low-efficiency filter with minimal efficiency removal values, MERV = 8; mid-efficiency filter, MERV = 12; and HEPA filter [43]), (ii) air filter use schedule (8-hour, 15-hour, and all-day), and (iii) smoking status of household member (yes or no). We ran CONTAM modeling across all combinations of the above dimensions, resulting in 20 simulations in total (Table S2). The air filter we modeled has a clean air delivery rate (CADR) of 200 cubic feet per minute (cfm), corresponding to 5.66 m^3^/min. The CADR rating system was used by American National Standards Institute (ANSI), indicating the volume of filtered air by an air-filtering device over time [48]. The portable air purifier of CADR 200 with HEPA filter represents the average level of dominant commercial air filters available on the Indian market; prices range between $250 and $1500 [23]. We also modeled air filters with lower efficiency to reflect lower-quality and more affordable air filter products. Table S3 shows the detailed microenvironmental locations of indoor occupants and user schedule of air filter. 

The CONTAM output files included PM_2.5_ concentrations in each room, PM_2.5_ personal exposure levels, and airflow rates into and out of each apartment wall, in hourly time increment over one year. 

### 2.5. Statistical Analysis

**Figure 3:**
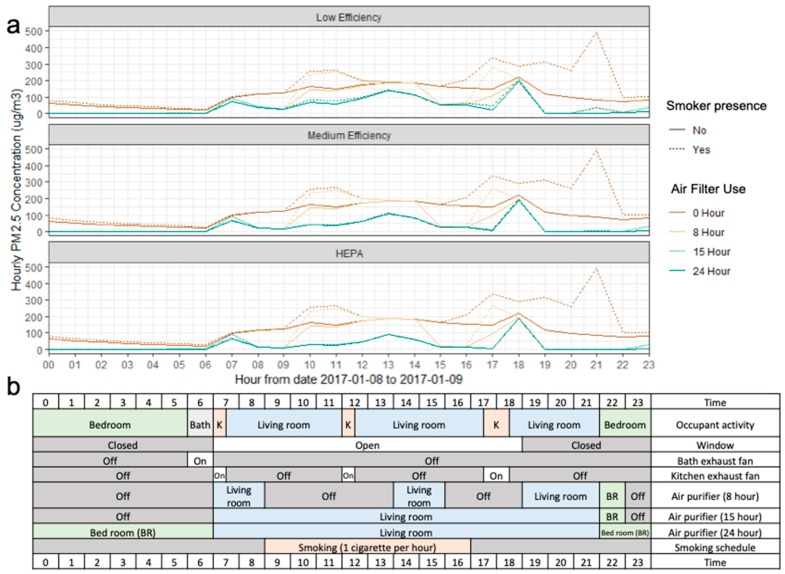
Illustration of CONTAM model output and data analysis from 8 January to 9 January 2017; (**a**) line plot of ambient PM_2.5_ and personal exposure to PM_2.5_ under different air filter efficiencies; (**b**) occupant schedule (K: kitchen, BR: bedroom).

We used the CONTAM Result Export Tool, an online data export tool [49], to convert CONTAM output files into txt and csv files. We used R (version 3.4, the R Foundation, Vienna, Austria) to analyze personal indoor PM_2.5_ exposure concentrations across all rooms in the apartment. Figure 3 shows the line plot of a one-day period of ambient PM_2.5_ concentrations and PM_2.5_ personal exposure during a heavily polluted day (9 January 2017) combined with occupant activities and air filter use schedules applied in simulations. We analyzed all year-round personal PM_2.5_ exposure derived from CONTAM models and assessed the reduction of annual PM_2.5_ personal exposure from air filter use.

### 2.6. Mortality Reduction Associated with Air Filter Use

To estimate mortality associated with indoor PM_2.5_ exposure and mortality associated with air filter use, we modeled mortality per 100,000 population over one year for a customized version of the Household Air Pollution Intervention Tool (HAPIT) [37]. Additional details on the methodology are available in the Supplemental Information, and it has been published elsewhere [37]. Briefly, HAPIT estimated averted death using standard Global Burden of Disease Methods and counted for five causes of death—chronic obstructive pulmonary disease, ischemic heart disease, stroke, and lung cancer (for all ages), and acute lower respiratory infection (ALRI) in those under five years old. The main modification to the current version of HAPIT was the utilization of sub-national background disease specific to Delhi generated as part of the 2016 GBD India Exercise [50] and calculation of benefits for a single year. We estimated averted mortality rates attributable to the scenarios outlined previously, with different air filter efficiencies, air filter uses patterns, and presence/absence of a smoker.

## 3. Results

**Figure 4:**
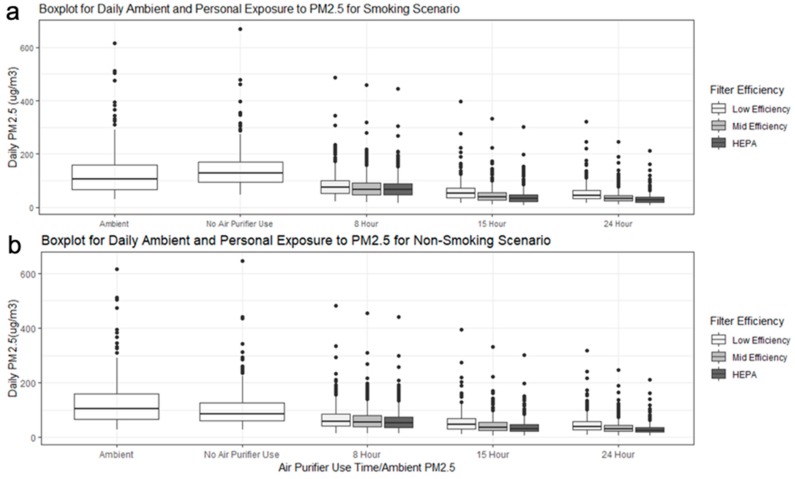
Boxplot for daily ambient PM_2.5_ concentrations and personal exposure at different air filter use schedules; (**a**) smoking, (**b**) non-smoking.

Figure 4 shows a boxplot for estimated daily ambient PM_2.5_ concentration and personal PM_2.5_ exposures for all 20 simulated scenarios over a year. The annual mean personal indoor PM_2.5_ exposure without smoking was 103 μg/m^3^ (95% CI: 93–112). It was lower than the annual ambient PM_2.5_ mean concentration of 123 μg/m^3^ (95% CI: 115–131). The annual mean personal indoor PM_2.5_ exposure with an active smoker was 137 μg/m^3^ (95% CI: 125–149), higher than the ambient PM_2.5_ concentration. Figure 4 also shows that with increasing PM_2.5_ removal efficiency and air filter use time, the annual mean personal PM_2.5_ exposure decreased in both smoking and non-smoking households.

Based on CONTAM simulations, the highest reduction of estimated personal PM_2.5_ exposure occurred in scenarios with all-day air filter use. However, only all-day use of HEPA filter yielded annual mean PM_2.5_ personal exposure levels below 35 μg/m^3^, the WHO Indoor Air Quality Guideline Interim Target 1 [7]. The 15-hour air filter use scenario also reduced air pollution exposure levels significantly, especially for HEPA filters, where the exposure level was approximately 39 μg/m^3^ (without a smoker) and 40 μg/m^3^ (with a smoker at home). Scenarios involving 8-hour air filter use did not perform as well as the others, even for HEPA filters; all the annual mean PM_2.5_ exposures with 15-hour and 8-hour air filter use exceeded the WHO Indoor Air Quality Guideline Interim 1 Target of 35 μg/m^3^. This may be due to the relatively high air exchange rate (annual mean AER = 1.5/h) of the whole apartment, leading to the infiltration of ambient air pollution to the indoor environment.

Table 3 shows CONTAM simulated annual mean personal indoor PM_2.5_ exposures as well as mortality averted for our scenarios with varied filter use and efficiency and the presence or absence of a smoker. Using air filters can reduce PM_2.5_ exposures dramatically, ranging from 31%–72% for smoker-absent scenarios and 38%–78% for smoker-present scenarios. From HAPIT, we estimated 698 deaths and 895 deaths per 100,000 person/year are associated with indoor PM_2.5_ exposure without air filter use in Delhi, for smoker-absent and smoker-present scenarios, respectively. Based on personal PM_2.5_ exposure reduction from air filter use, we estimated that using an air filter all day can reduce mortality associated with indoor air pollution by between 8% and 37%.

In our sensitivity analyses, we evaluated the influence of window open time, window size, floor level, and time spent outdoors on the effectiveness of air filters (Table S4). We found that with increased duration of windows open and increased window area, annual mean AERs increased from 1.5/h to 3.3/h when windows with 1 m^2^ cross-sectional area were opened for 24 hours per day, and the effectiveness of the air filter decreased. Under that scenario, the annual mean PM_2.5_ exposure increased from 29 μg/m^3^ to 58 μg/m^3^, mainly due the infiltration of ambient air pollution from the outdoor environment. When increasing the duration of time spent outdoors by 2 hours (from 14:00–16:00), we also found an increase in annual PM_2.5_ personal exposure and decrease in effectiveness of air filters. The floor of the apartment building did not significantly influence PM_2.5_ exposure, with less than 5% difference in AERs and annual mean PM_2.5_ exposures. This is partly due to the fact that CONTAM simulations did not show large difference in AERs across first and fourth floor apartments, leading to similar PM_2.5_ personal exposure levels.

## 4. Discussion

We simulated use of an air filter in a residential household in urban Delhi for one year with the CONTAM program and estimated its impact on the reduction of personal exposure to PM_2.5_ from indoor and outdoor origins for one occupant living at home. We also estimated the effect on mortality from different air filter use scenarios through PM_2.5_ exposure reduction. This is the first study modeling the effectiveness of air filter use in developing countries. Results suggest that using air filters can achieve substantial reductions in air pollution exposures, and these reductions could avert significant ill-health associated with air pollution. The protective effects of the filter are greater with increased use of higher quality, high-efficiency filters.

Our simulations are based on the CONTAM modeling program that makes certain assumptions on indoor particle dynamics and on an occupant’s schedule over a year. Previous evaluations of CONTAM have shown that its simulations of airflow, AERs, and particle concentrations were in good agreement with field measurements [51,52]. In our CONTAM simulations, airflow across each room and in/out of each room was calculated hourly, as were indoor PM_2.5_ concentrations under a well-mixed microenvironment assumption. Our CONTAM simulation has an annual mean AER of 1.5/h (daily range 0.3 to 4.5/h) for residential homes in Delhi, which is smaller than the previously measured AER of 2.5/h–5.1/h from urban roadside homes near Agra, India [53]. Our simulated AER was also based on the assumption that the occupants would adjust their behaviors to close windows at night and reduce air exchange when the air filter was in use, leading to smaller AERs compared to the scenarios when windows are always open in previous studies [53]. Nevertheless, the AER of 1.5/h is still considered very high in urban homes in United States [31], and this also causes a relatively high infiltration of outdoor air pollution to the indoor environment. In the sensitivity analysis, we conducted simulations to allow larger window areas and longer periods with the windows open. The AERs increased to 3.3/h annual mean (daily range 0.5/h–11.1/h) when windows with 1 m^2^ cross-sectional area are open for 24 hours per day, and annual PM_2.5_ exposure levels with all-day use of HEPA air filter increased to 58 μg/m^3^, indicating considerable reduction in air filter effectiveness (Table S4).

The indoor PM_2.5_ exposure results from our study are consistent with prior studies that investigated indoor air filter use and air pollutant exposure, either by modeling [31,34] or field measurement [20,22,23,24]. Our study adds to the current evidence base by modeling the effects of air filters with different efficiencies and various user scenarios for a year, as well as by estimating mortality reduction. The relatively small health benefits, compared to larger PM_2.5_ exposure reduction, are mainly due to the shape of the state-of-the-science exposure-response curve used in HAPIT tool [37], indicating further reduction of PM_2.5_ exposure level is needed to achieve more substantial public health benefits. When the air filter is used all day, we found little difference in personal exposure to PM_2.5_ between smoker-absent and smoker-present scenarios (29 μg/m^3^ vs. 30 μg/m^3^). This implies minimal offset of health risks from passive smoking by using indoor air filters. This also suggests that outdoor PM_2.5_ infiltration may have more influence on personal exposure than other factors, including the presence of a smoker in the home. Indoor smoking in homes, however, poses numerous other risks and, naturally, use of an air filter would not abate exposures to the smoker and likely would reduce but not eliminate second-and-thirdhand smoke exposures to individuals in proximity of the smoker. 

As these results are derived from hypothetical intensive air filter use, they may not fully represent real-life situations. Our model indicated 15-hour and 8-hour air filter use cannot reduce PM_2.5_ below 35 μg/m^3^. This limited exposure and health risk reduction capacity was mainly due to the relatively high AER (1.5/h) and high ambient air pollution in Delhi. Considering the fact that many residential buildings have AER higher than 1.5/h, [53] the effectiveness of air filters could be further diminished if the buildings have more leaks or windows are open for longer periods of time, as indicated in our sensitivity analysis (Table S4). Therefore, we believe that air filters may not be considered as the sole intervention strategy to reduce health risk from indoor air pollution exposure; other interventions and exposure reduction strategies targeting the sources and transport of high-level ambient air pollution in Delhi should also be in place [23]. Additionally, the price range of dominant commercial air filters on the Indian market is $250–$1500. Air filter is still a costly home appliance compared to the average monthly expenditure of urban Delhi residents [23]. Therefore, cost–benefit analyses for air filter use could be another important future research direction to maximize the potential of air filter use in combating this pressing environmental health issue. 

A limitation of this study is that we did not conduct field investigations (personal PM_2.5_ exposure measurement and population epidemiological studies) to validate the assumptions or results. Thus, while this study is suggestive of potential reductions in exposure and associated health benefits, these must to confirmed in the field. One such study is underway in Ulaanbaatar, Mongolia, where wintertime ambient PM_2.5_ concentrations often exceed those of Delhi [54,55,56]. Another limitation of this study is that some of our assumptions may not fully reflect the building environment in urban Delhi and the variabilities in population behaviors, due to lack of building stock data and population time–activity pattern data. Though we conducted sensitivity analysis to model the effectiveness of air filter use in apartments with different window open schedules, AERs, and occupant schedules, there will often be gaps between simulation results and actual effectiveness assessment of air filter use in real urban Delhi homes.

## 5. Conclusions

Our simulation suggests that consistent use of indoor air filters can reduce indoor air pollution exposure for urban Delhi households. The reduced exposure from air filtration could avert between 8% and 37% of air pollution related mortality, depending on air filter efficiency, use time, and passive smoking behaviors. If these results were confirmed experimentally, air filters could offer significant health benefits to residents of highly polluted urban environments.

## Figures and Tables

**Table 1:** Household characteristics inputs for CONTAM simulations.

Model Input Parameter	Parameter Description	Schedule	Reference
Floor plan	Apartment containing 1 living room, kitchen, bathroom, and bedroom, 30 m^2^		Residential buildings in India: energy use and saving potentials, Global building performance network, 2014 [38]
Wall leakage	Wall leakage area 5 cm^2^/m^2^		Residential buildings in India: energy use and saving potentials, Global building performance network, 2014 [38]
Window	0.8 m^2^ open area in total	Open: 7:00–18:00	
Bath exhaust fan	120 m^3^/h (70 cfm †)	On: 6:00–7:00	Fabian et al., 2011, Indoor Air [31]
Kitchen exhaust fan	170 m^3^/h (100 cfm)	On when cooking (7:00–7:30; 12:00–12:30; 17:00–18:00)	Fabian et al., 2011, Indoor Air [31]

† cubic feet per minute.

**Table 2:** Indoor fine particulate matter (PM_2.5_) sources, sinks, emission/removal rates, air filter PM_2.5_ removal efficiency, weather, and ambient air pollution data used in CONTAM simulation.

Source/Sink and Parameter	Emission/Removal Rate	Schedule	Source
Cooking	+0.14 mg/min	2 h a day	
7:00–7:30; 12:00–12:30; 17:00–18:00	Shen et al., 2018, Environmental Science and Technology [39]		
Smoking	+0.33 mg/min	8 cigarettes per day, one per hour from 9:00–14:00 in the day time	Fabian et al., 2011, Indoor Air [31]
PM_2.5_ deposition	−0.19/h		Fabian et al., 2011, Indoor Air [31]
Air Filter at 200 Clean Air Delivery Rate (CADR), PM_2.5_ removal efficiency	HEPA filter: 0.99	Either 8 h, 15 h, or 24 h a day	Azimi et al., 2014, Atmospheric Environment [44]
	Medium efficiency filter: 0.65		
	Low efficiency filter: 0.3		
Weather			Typical meteorological year (TMY) hourly weather data from Energy Plus [45]

**Table 3:** Annual mean exposure to PM_2.5_ indoors and health benefits due to air filter use at different scenarios from CONTAM program.

		Annual Mean PM_2.5_ Exposure (μg/m^3^)		Mortality (% of Avoidable †) Averted per Million People	
		Smoker Absent	Smoker Present	Smoker Absent	Smoker Present
8-hour Air Filter use	No Air Filter	103	137	NA	NA
	Low efficiency filter	71	84	61 (8.3)	75 (10.2)
	Mid efficiency filter	65	77	77 (10.3)	90 (12.1)
	HEPA filter	62	78	85 (11.5)	88 (11.9)
15-hour Air filter use	Low efficiency filter	56	60	104 (14)	134 (18.1)
	Mid efficiency filter	44	46	150 (20.2)	184 (24.8)
	HEPA filter	39	40	174 (23.5)	212 (28.6)
All day air filter use	Low efficiency filter	50	56	125 (16.9)	146 (19.8)
	Mid efficiency filter	35	38	196 (26.5)	222 (30)
	HEPA filter	29	30	235 (31.8)	271 (36.6)

† Percent of the total air pollution burden in Delhi avoided by the intervention in one year.

## Supplementary Materials

The following are available online at https://www.mdpi.com/1660-4601/16/8/1391/s1, Figure S1: Map of study area and ambient air monitoring stations; Table S1: Ambient air pollution station and corresponding longitude and latitude; Table S2: Details of CONTAM simulation models; Table S3: Microenvironment schedule of occupants and user schedule for air filter; Table S4: Sensitivity analysis with different window opening times, window sizes, floors, and time spent outdoors.
