# Supplementary material for: Modeling the Impact of an Indoor Air Filter on Air Pollution Exposure Reduction and Associated Mortality in Urban Delhi Household
Source: Int J Environ Res Public Health. 2019 Apr 17;16(8):1391. doi: 10.3390/ijerph16081391 (PMC6518106; doi:10.3390/ijerph16081391)
Supplement: Supplementary file 1 [file ijerph-16-01391-s001.pdf]

*Article*

# Modeling the Impact of an Indoor Air Filter on Air Pollution Exposure Reduction and Associated Mortality in Urban Delhi Household

**Jiawen Liao [1,*,^], Wenlu Ye [1,^], Ajay Pillarisetti [2] and Thomas F. Clasen [1]**

[1] Department of Environmental Health, Rollins School of Public Health, Emory University, Atlanta, GA, 30322, United States; wenlu.ye@emory.edu (W.Y.); thomas.f.clasen@emory.edu (T.F.C.)

[2] Environmental Health Sciences, School of Public Health, University of California, Berkeley, CA 94720, United States; ajaypillarisetti@berkeley.edu

* Correspondence: jiawen.liao@emory.edu

^ Both authors contributed equally to the study.

## Supplementary Information

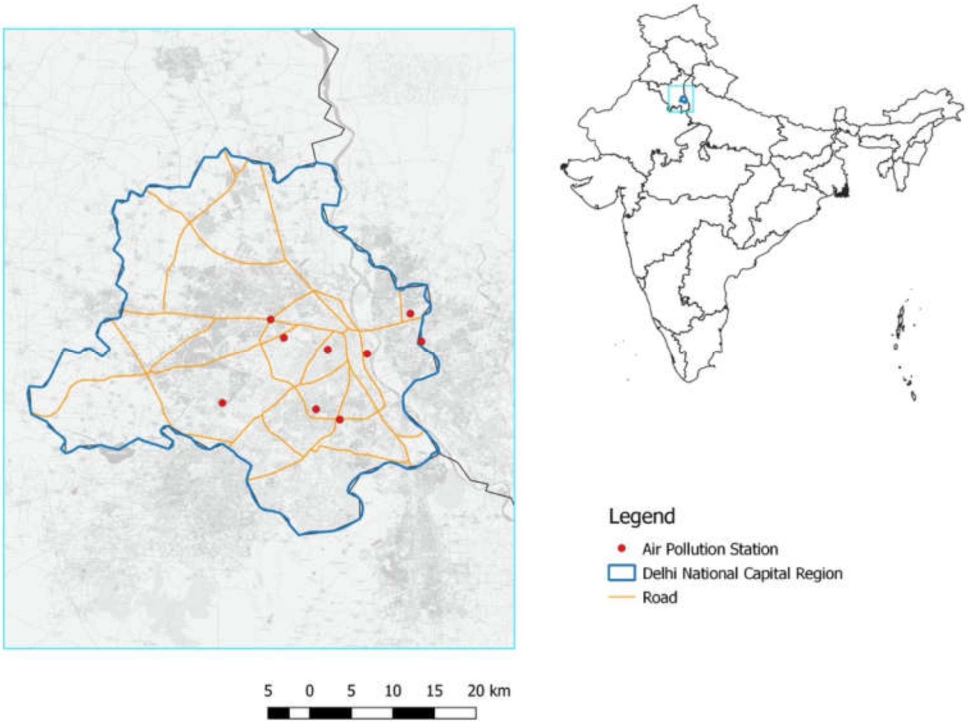


**Figure S1.** Map of study area and ambient air monitoring stations.

**Table S1.** Ambient air pollution station and corresponding longitude and latitude.

| Station Name | Longitude | Latitude |
|---|---|---|
| Anand Vihar | 77.316032 | 28.646835 |
| Dwarka | 77.0719006 | 28.5710274 |
| IHBAS | 77.3025234 | 28.6811736 |
| ITO | 77.2494387 | 28.6316945 |
| Mandir Marg | 77.201067 | 28.636429 |
| Punjabi Bagh | 77.186937 | 28.563262 |

| | | |
|---|---|---|
| RK Puram | 77.131023 | 28.674045 |
| Shadipur | 77.1473105 | 28.6514781 |
| Siri Fort | 77.2159377 | 28.5504249 |

**Table S2.** Details of CONTAM simulation models.

| Model Number | Filter $PM_{2.5}$ Removal Efficiency (% $PM_{2.5}$ removal) | Air Filter Use (hours/day) | Smoker Presence |
|---|---|---|---|
| 1 | HEPA (99%) | 8 | No |
| 2 | HEPA (99%) | 15 | No |
| 3 | HEPA (99%) | 24 | No |
| 4 | Medium efficiency (65%) | 8 | No |
| 5 | Medium efficiency (65%) | 15 | No |
| 6 | Medium efficiency (65%) | 24 | No |
| 7 | Low efficiency (30%) | 8 | No |
| 8 | Low efficiency (30%) | 15 | No |
| 9 | Low efficiency (30%) | 24 | No |
| 10 | HEPA (99%) | 8 | Yes |
| 11 | HEPA (99%) | 15 | Yes |
| 12 | HEPA (99%) | 24 | Yes |
| 13 | Medium efficiency (65%) | 8 | Yes |
| 14 | Medium efficiency (65%) | 15 | Yes |
| 15 | Medium efficiency (65%) | 24 | Yes |
| 16 | Low efficiency (30%) | 8 | Yes |
| 17 | Low efficiency (30%) | 15 | Yes |
| 18 | Low efficiency (30%) | 24 | Yes |
| 19 | No filter Use | 0 | Yes |
| 20 | No filter use | 0 | No |

**Table S3.** Microenvironment schedule of occupants and user schedule for air filter.

| Schedule and Location | |
|---|---|
| 8-hour air filter use | Living room: 7:00–9:00, 14:00–16:00, 19:00–21:00; Bedroom: 21:00–22:00 |
| 15-hour air filter use | Living room: 7:00–21:00; Bedroom: 21:00–22:00 |
| All day air filter use | Living room: 7:00–21:00; Bedroom: 21:00–7:00 (next day) |
| Occupants schedule | Bathroom: 6:00–7:00, Kitchen: 7:00–7:30, 12–12:30, 17:30–18:30; Living room: 7:30–12:00, 12:30–17:30, 18:30–22:00; Bedroom: 21:00–7:00; |

**Table S4.** Sensitivity analysis with different window opening times, window sizes, floors, and time spent outdoors.

| Simulation Inputs Modified for Sensitivity Analysis | Daily AER (/h), min–max (mean) | Annual Mean $PM_{2.5}$ Exposure (μg/m$^3$) |
|---|---|---|
| Original Model [1] | 0.3–4.5 (1.5) | 29 |
| 17 hours of windows open per day (7:00–24:00) | 0.3–5.2 (1.7) | 37 |
| 24 hours of windows open per day (0:00–24:00) | 0.4–6.3 (1.9) | 43 |
| 24 hours of windows open per day (0:00–24:00) and 1 m$^2$ windows cross-sectional opening area | 0.5–11.1 (3.3) | 58 |
| 4th floor | 0.3–4.7 (1.5) | 30 |
| 2 hours of outdoor activity per day (14:00–16:00) | 0.3–4.5 (1.5) | 38 |

[1] 24 h HEPA filter use, without smoker 11 hours of window open per day (7:00–18:00), 0.8 m$^2$ windows cross-sectional opening area, 1st floor apartment, all-day indoor occupant schedule.

**Estimating the burden averted due to various air filtration scenarios**

Estimates of averted mortality were calculated using standard Global Burden of Disease Methods [1]. Background disease data for Delhi were manually extracted from the IHME GBD Compare India website [2] for five causes of death—chronic obstructive pulmonary disease, ischemic heart disease, stroke, and lung cancer (for all ages), and ALRI in those under five years old. Extracting chronic disease endpoints for adults only was not possible, as age categories overlapped. Cases in ages under 20 represented a small number (less than 0.30%) of the mortality for each of these diseases.

For each of the CONTAM-modeled exposures, relative risks were derived from curves generated using publicly available parameters provided by IHME and described in Burnett et al. [3]. Population attributable fractions (PAFs) were estimated and pre- and post-intervention burdens were calculated by multiplying the disease specific PAF by the mortality attributed to each background disease. The post-intervention disease burden was subtracted from the pre-intervention burden to estimate the averted burden for each scenario.

**References**

1. Smith, K.R.; Bruce, N.; Balakrishnan, K.; Adair-Rohani, H.; Balmes, J.; Chafe, Z.; Dherani, M.; Hosgood, H.D.; Mehta, S.; Pope, D.; et al. Millions Dead: How Do We Know and What Does It Mean? Methods Used in the Comparative Risk Assessment of Household Air Pollution. *Annu. Rev. Public Health* **2014**, *35*, 185–206.
2. ICMR; PHFI; IHME Indian Council of Medical Research, Public Health Foundation of India, and Institute for Health Metrics and Evaluation Available online: http://vizhub.healthdata.org/gbd-compare/india (accessed on 6 December 2018).
3. Burnett, R.T.; Pope, C.A., III; Ezzati, M.; Olives, C.; Lim, S.S.; Mehta, S.; Shin, H.H.; Singh, G.; Hubbell, B.; Brauer, M.; et al. An Integrated Risk Function for Estimating the Global Burden of Disease Attributable to Ambient Fine Particulate Matter Exposure. *Environ. Health Perspect.* **2014**.
